# Total Cerebral Small Vessel Disease MRI Score Is Associated with Cognitive Decline in Executive Function in Patients with Hypertension

**DOI:** 10.3389/fnagi.2016.00301

**Published:** 2016-12-12

**Authors:** Renske Uiterwijk, Robert J. van Oostenbrugge, Marjolein Huijts, Peter W. De Leeuw, Abraham A. Kroon, Julie Staals

**Affiliations:** ^1^Department of Neurology, Maastricht University Medical CentreMaastricht, Netherlands; ^2^School for Mental Health and Neuroscience, Maastricht UniversityMaastricht, Netherlands; ^3^Cardiovascular Research Institute Maastricht, Maastricht UniversityMaastricht, Netherlands; ^4^Department of Psychiatry and Psychology, Maastricht University Medical CentreMaastricht, Netherlands; ^5^Department of Internal Medicine, Maastricht University Medical CentreMaastricht, Netherlands

**Keywords:** Small vessel disease, cognitive decline, executive functioning, hypertension, white matter hyperintensities, cerebral microbleeds, lacunes, perivascular spaces

## Abstract

**Objectives:** Hypertension is a major risk factor for white matter hyperintensities (WMH), lacunes, cerebral microbleeds, and perivascular spaces, which are MRI markers of cerebral small vessel disease (SVD). Studies have shown associations between these individual MRI markers and cognitive functioning and decline. Recently, a “total SVD score” was proposed in which the different MRI markers were combined into one measure of SVD, to capture total SVD-related brain damage. We investigated if this SVD score was associated with cognitive decline over 4 years in patients with hypertension.

**Methods:** In this longitudinal cohort study, 130 hypertensive patients (91 patients with uncomplicated hypertension and 39 hypertensive patients with a lacunar stroke) were included. They underwent a neuropsychological assessment at baseline and after 4 years. The presence of WMH, lacunes, cerebral microbleeds, and perivascular spaces were rated on baseline MRI. Presence of each individual marker was added to calculate the total SVD score (range 0–4) in each patient.

**Results:** Uncorrected linear regression analyses showed associations between SVD score and decline in overall cognition (*p* = 0.017), executive functioning (*p* < 0.001) and information processing speed (*p* = 0.037), but not with memory (*p* = 0.911). The association between SVD score and decline in overall cognition and executive function remained significant after adjustment for age, sex, education, anxiety and depression score, potential vascular risk factors, patient group, and baseline cognitive performance.

**Conclusion:** Our study shows that a total SVD score can predict cognitive decline, specifically in executive function, over 4 years in hypertensive patients. This emphasizes the importance of considering total brain damage due to SVD.

## Introduction

Hypertension is a major risk factor for brain damage such as white matter hyperintensities (WMH), lacunes, cerebral microbleeds, and perivascular spaces (Veglio et al., 2009). These MRI markers of brain damage result from small vessel disease (SVD). Several cross-sectional studies have investigated the implications of these MRI markers on cognition and showed significant associations with lower cognitive functioning or cognitive impairment (de Groot et al., 2000; van der Flier et al., 2005; Pantoni et al., 2007; Carey et al., 2008). Also in longitudinal research, associations between decline in cognitive functioning and WMH and lacunes have been found (Vermeer et al., 2003; Prins et al., 2005; Jokinen et al., 2011; Pavlovic et al., 2014). Studies in cerebral microbleeds and perivascular spaces are more sparse, but a few studies have reported that these might also be related to cognitive decline or a higher risk of incident dementia (Zhu et al., 2010; Gregoire et al., 2012).

These previous studies mostly focused on the different MRI markers of SVD separately. There are some studies investigating the combined effect of WMH and lacunes on cognitive function or decline (Baune et al., 2009; Jokinen et al., 2011), but no study combined all MRI markers. Recently, a “total SVD score” was proposed to combine all individual MRI markers into one measure of SVD, which aims to capture total brain damage from SVD (Staals et al., 2014). This total SVD score was found to be associated with higher blood pressure levels (Klarenbeek et al., 2013b), hypertension, age, and other risk factors for SVD (Staals et al., 2014). Two recent cross-sectional studies have shown that this total SVD score was related to lower cognitive performance (Huijts et al., 2013; Staals et al., 2015). However, the SVD score, combining all four markers, has never been applied in longitudinal research, studying cognitive decline.

The aim of the present study was to investigate if the SVD score was associated with cognitive decline over 4 years in hypertensive patients. To compose a patient cohort with a broad spectrum of severity of cerebral SVD, we included patients with uncomplicated hypertension as well as hypertensive patients with a prior clinical lacunar stroke. In addition, we examined if each of the MRI markers in this SVD score individually contributed to cognitive decline, in which it was expected that WMH make the largest contribution to cognitive decline

## Materials and Methods

### Study Population

We included patients with hypertension, who were selected from two larger studies: the hypertension and brain damage study (HYBRiD) (Henskens et al., 2008) and the cognitive function in small vessel stroke study (Huijts et al., 2013).

In HYBRiD, essential hypertensive patients were recruited from the hypertension outpatient clinic of the Department of Internal Medicine of Maastricht University Medical Centre, the Netherlands. Hypertension was defined as an off-medication, clinically measured conventional blood pressure ≥140 mm Hg systolic and/or ≥90 mm Hg diastolic. Exclusion criteria were a history of symptomatic cardio- or cerebrovascular disease or contraindications for MRI. Details about the HYBRiD study have been described before (Henskens et al., 2008). A total of 218 patients were included in the HYBRiD study and they were asked to participate in the present study.

In the cognitive function in small vessel stroke study, first-ever lacunar stroke patients were recruited from the Neurology Department of the Maastricht University Medical Centre between February 2009 and July 2012. Lacunar stroke was defined as an acute stroke syndrome with a small (<20mm) ischemic lesion on acute brain MR in the brain stem, basal ganglia or internal capsule, compatible with the occlusion of a single perforating small artery. If no such lesion was visible, we used established clinical criteria for lacunar stroke (De Jong et al., 2002). Patients with severe comorbidity, either neurological or psychiatric, were excluded. Furthermore, patients with possible other causes for the lacunar stroke than cerebral SVD (cardiac embolic source, cerebral large vessel disease, or carotid stenosis), were also excluded (Huijts et al., 2013). A total of 77 patients were included in the small vessel stroke study, and those who had hypertension (defined as the use of antihypertensive medication or a history of hypertension) were selected for the present analysis.

All patients underwent a brain MRI scan and a neuropsychological assessment. For hypertensive lacunar stroke patients, the neuropsychological assessment was performed 3 months after stroke to exclude acute phase effects. The neuropsychological assessment was repeated 4 years later. Registration of educational level was based on the Dutch classification system “Verhage” [in which levels 1, 2, 3, and 4 are considered as low education, level 5 is considered as middle education and levels 6 and 7 are considered as high education (Verhage, 1964)]. Information about vascular risk factors [body mass index (BMI), smoking, and the presence of diabetes mellitus or hypercholesterolemia] was obtained based on patient self-report.

The Medical Ethics Committee of the Maastricht University Medical Centre approved this study and all participants gave written informed consent.

### MRI Data

All HYBRiD patients were scanned on a 1.5T MRI scanner, while the lacunar stroke patients were scanned using a 1.5T or a 3T scanner (both scanners: Philips Medical Systems, Best, The Netherlands). For lacunar stroke patients, the median time between stroke and MRI was 6 days (IQR = 3.5–32.5). On brain MRI scans (standard axial T2-weighted, FLAIR and T2^∗^ gradient echo sequences, details are described in Supplementary Data Sheet) two experienced vascular neurologists individually rated the markers of SVD. The inter-rater agreement statistics have been previously reported (Huijts et al., 2013). An ordinal scale representing the total burden of SVD was created. Definitions and rating method have been described in detail before (Staals et al., 2014). In short, the presence of each of the four MRI markers for SVD (WMH, lacunes, cerebral microbleeds and perivascular spaces) was counted to retrieve a total SVD score (ranging 0–4). WMH were assessed according to the Fazekas scale (Fazekas et al., 1987). Presence of WMH was defined as periventricular WMH Fazekas score 3 (irregular hyperintensities extending into the deep white matter) and/or deep WMH Fazekas score 2 or 3 (confluent hyperintensities). Lacunes and cerebral microbleeds were assessed according to the international consensus definition (Wardlaw et al., 2013) and for both lacunes and cerebral microbleeds, one point was awarded in the total SVD score if at least one lacune or cerebral microbleed was present. The recent symptomatic small subcortical infarct in the lacunar stroke patients was not counted as a lacune, because the SVD score intends to rate background SVD burden. Perivascular spaces were rated as mild, moderate or extensive at the level of the basal ganglia, since perivascular spaces in the basal ganglia are specifically related to SVD (Doubal et al., 2010; Potter et al., 2015). One point was awarded for moderate or extensive perivascular spaces in the basal ganglia.

### Neuropsychological Assessment

Cognitive performance was measured with a comprehensive neuropsychological assessment at baseline and after 4 years of follow-up, as has been described before (Uiterwijk et al., 2014). The test protocol was identical in both patient groups. Memory domain was measured with the Rey Auditory Verbal Learning Test (Brand and Jolles, 1985) (immediate recall, delayed recall and delayed recognition) and the Digit Span Forward [subtest of Wechsler Adult Intelligence Scale (WAIS)-III (Wechsler, 2001)]. Executive function domain was measured with the Stroop Color Word Test (Golden, 1978) (SCWT) interference score (time of part 3minus mean time of parts 1 and 2), Trail Making Test (TMT) (Reitan, 1956) interference score (time of part B minus time of part A), Category (animals and professions) (Luteyn, 1966) and Letter Fluency (Lezak et al., 2004), Letter-Number Sequencing (subtest of WAIS-III), and Digit Span Backward (subtest of WAIS-III). Information processing speed domain was measured with the Symbol Substitution - Coding (subtest of WAIS-III), TMT part A, and SCWT parts 1 and 2. Parallel versions were used for baseline and follow up assessment for the Rey Auditory Verbal Learning Test and the Letter Fluency test.

For each patient, we subtracted the raw test scores at follow-up from test scores at baseline. These raw test decline scores were transformed into standardized values (z-scores), by dividing the difference between the individual raw score and the overall group sample mean by the overall group sample standard deviation (SD). Z-scores of all tests within one domain were averaged to receive cognitive decline compound scores of each domain. In addition, an overall cognitive decline compound score was calculated by averaging the three domain compound scores. Higher compound scores indicated higher decline. Z-scores of tests with higher scores representing worse performance (e.g., SCWT and TMT) were inverted before computing the compound scores.

The Rotterdam - Cambridge Cognitive Examination (R-CAMCOG) was used to determine the presence of possible dementia, defined as a score < 34 (de Koning et al., 2005). Symptoms of depression and anxiety were measured using the Hospital Anxiety and Depression Scale (HADS) and the total score (range 0–42) was used. Because symptoms possibly caused by physical problems (e.g., insomnia or weight loss) are not included in the HADS, the scale is considered to be suitable to use in somatic populations (Spinhoven et al., 1997).

### Statistical Analysis

Differences in baseline characteristics between uncomplicated hypertensive patients and hypertensive lacunar stroke patients were investigated using independent *t*-test, chi-square test or Mann–Whitney *U* test. The associations between SVD score and age, sex, HADS total score, low or high educational level, and vascular risk factors were examined using spearman’s correlation analysis or Mann–Whitney *U* test. Associations between the SVD score and cognitive decline (overall cognition and separate domains of executive functioning, information processing speed and memory) were investigated using simple linear regression analyses. Next, the association between SVD score and cognitive decline was adjusted for possible confounders in three models of multivariable linear regression analyses. In model 1, the associations between SVD score and cognitive decline scores were adjusted for age, sex, educational level, HADS total score, and potential confounding vascular risk factors (BMI, smoking, and the presence of diabetes mellitus or hypercholesterolemia). To avoid overfitting, we only included those vascular risk factors that were associated with cognitive decline with *p* < 0.10 in a simple regression model. In model 2, we corrected for the occurrence of a symptomatic stroke, by adding patient group (patient with uncomplicated hypertension or hypertensive lacunar stroke patient) to the previous model. In model 3, baseline cognitive performance (of the corresponding cognitive domain) was added to model 2.

Coefficients of determinations (*R*^2^) were calculated in simple linear regression analyses to determine the proportion of variance in cognitive decline explained by the SVD score. To investigate the contribution of each of the markers separately, we repeated the analyses with WMH, lacunes, microbleeds and perivascular spaces as predictor individually (dichotomised, as they were defined in the SVD score) and *R*^2^’s were calculated. In addition, *R*^2^ of the full WMH Fazekas score (deep and periventricular scores summed, score ranging 0–6) was calculated.

IBM SPSS Statistics 22 software was used for all analyses. Results were considered significant at *p* < 0.05.

## Results

### Participants

**FIGURE 1:**
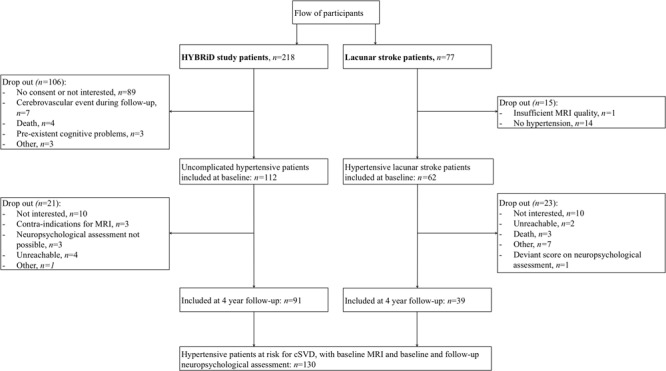
**Participant flow diagram**.

Flow of participants is shown in **Figure 1**. We included 112 HYBRiD hypertensive patients and 62 hypertensive lacunar stroke patients in the present study at baseline. Of these, 91 hypertensive patients and 39 lacunar stroke patients completed 4-year follow-up, giving 130 patients in this study. The mean follow-up period was 4.04 year (*SD* = 0.10). Included patients (*n* = 130) did not differ from the excluded patients (*n* = 165) in age (58.7 ± 12.2 *vs*. 59.3 ± 13.5 years, respectively, *p* = 0.70) or sex (male 57.7% *vs*. 47.0%, respectively, *p* = 0.09). Patients who completed follow-up (*n* = 130) did not differ from patients who only completed baseline (*n* = 44) regarding sex (male 57.7% *vs*. 59.1%, respectively, *p* = 0.87), but were younger (58.7 ± 12.1 *vs*. 65.1 ± 13.9, respectively, *p* = 0.004) and had lower baseline overall cognition compound scores (*p* = 0.012). Baseline characteristics, including educational level, HADS score and vascular risk factors, for the overall group and separately for uncomplicated hypertensive patients and lacunar stroke patients are shown in **Table 1**. Hypertensive lacunar stroke patients were older (*p* = 0.001), more often had hypercholesterolemia (*p* < 0.001), more often smoked (*p* = 0.02), had lower educational levels (*p* = 0.03), and had higher SVD scores compared to uncomplicated hypertensive patients (*p* < 0.001). Other baseline characteristics did not differ between uncomplicated hypertensive patients and lacunar stroke patients. Hypertensive lacunar stroke patients had a lower overall cognition score at baseline compared to uncomplicated hypertensive patients (*p* < 0.001), but there were no differences in cognitive decline between patient groups (*p* = 0.707). Three lacunar stroke patients had a recurrent stroke during follow-up, while none of the uncomplicated hypertensive patients had a stroke during follow-up.

**Table 1:** Patients’ characteristics for uncomplicated hypertensive patients and hypertensive lacunar stroke patients.

Baseline characteristic	All patients (*n* = 130)	Uncomplicated hypertensive patients (*n* = 91)	Hypertensive lacunar stroke patients (*n* = 39)
Age, mean (*SD*), years	58.7 (12.2)	56.3 (11.9)	64.2 (11.1)
Male sex, No. (%)	75 (57.7%)	52 (57.1%)	23 (59.0%)
Educational level, No. (%):			
– Low	42 (32.3%)	23 (25.3%)	19 (48.7%)
– Average	42 (32.3%)	33 (36.3%)	9 (23.1%)
– High	46 (35.4%)	35 (38.5%)	11 (28.2%)
HADS total score, median (IQR)	8 (4–13)	7 (4–12)	9 (4–14)
Diabetes Mellitus, No. (%)	3 (2.3%)	1 (1.1%)	2 (5.1%)
Hypercholesterolemia, No. (%)^∗^	57 (44.9%)	31 (34.8%)	26 (68.4%)
Current smoking, No. (%)	27 (20.8%)	14 (15.4%)	13 (33.3%)
BMI, mean (*SD)*, kg/m^2^	27.3 (4.2)	27.4 (4.2)	27.0 (4.0)
SVD score 0, No. (%)	63 (48.5%)	50 (54.9%)	13 (33.3%)
SVD score 1, No. (%)	33 (25.4%)	27 (29.7%)	6 (15.4%)
SVD score 2, No. (%)	18 (13.8%)	8 (8.8%)	10 (25.6%)
SVD score 3, No. (%)	13 (10.0%)	6 (6.6%)	7 (17.9%)
SVD score 4, No. (%)	3 (2.3%)	0 (0.0%)	3 (7.7%)

*SD = Standard deviation, HADS = Hospital Anxiety and Depression Scale, IQR = Interquartile range, BMI = Body Mass Index, SVD = Small vessel disease. ^∗^Information about hypercholesterolemia was missing for 3 patients (2 uncomplicated hypertensive patients and 1 hypertensive lacunar stroke patient).*

For two patients, one baseline neuropsychological test score in the domain of executive function was missing. Therefore, for these patients the executive function decline score was composed of the remaining 5 tests in this domain. For three patients, the baseline Rey Auditory Verbal Learning Test was missing. Since this test determines 3 out of 4 test scores of the memory domain, no reliable memory decline score could be formed for these two patients and consequently the overall cognition decline score was also missing. As a result, analyses of executive function and information processing speed decline are based on the data of 130 patients, while analyses of overall cognition and memory decline are based on the data of 127 patients.

### SVD Score

Presence of each category of the SVD score is shown in **Table 1**. The SVD score correlated with age (*p* < 0.001) and lower BMI (*p* = 0.043), but there were no significant differences in sex, educational level, HADS total score, or vascular risk factors.

### SVD Score and Cognitive Decline

At baseline three patients (2%; 1 uncomplicated hypertensive patient and 2 lacunar stroke patients) had an R-CAMCOG score <34, indicating possible dementia. At follow-up, besides these three patients, six (5%) other patients had a score <34. Three of these patients were uncomplicated hypertensive patients and six were hypertensive patients with a lacunar stroke (*p* = 0.021).

Unadjusted analyses showed an association between SVD score and cognitive decline in overall cognition, executive functioning and information processing speed, but not with memory (**Table 2**). The association between SVD score and decline in executive function remained significant after adjustment for age, sex, educational level, HADS score, and potential vascular risk factors (**Table 2**, model 1). Additional adjustment for patient group with or without additional adjustment for baseline cognition (of the corresponding cognitive domain) showed significant associations between SVD score and decline in overall cognition and executive function (**Table 2**, models 2 and 3).

**Table 2:** Association between small vessel disease (SVD) score and cognitive decline.

	Decline in overall cognition; *B* (95%CI)	*p*	Decline in executive function; *B* (95%CI)	*p*	Decline in information processing speed; *B* (95%CI)	*p*	Decline in memory; *B* (95%CI)	*p*
Simple regression analysis	0.08 (0.02–0.15)	0.017	0.17 (0.09–0.24)	<0.001	0.11 (0.007–0.22)	0.037	–0.006 (-0.11–0.10)	0.911
Multivariable regression analysis model 1	0.07 (-0.01–0.14)	0.072	0.13^∗^ (0.05–0.21)	0.003	0.08 (-0.04–0.20)	0.179	0.001 (-0.11–0.12)	0.984
Multivariable regression analysis model 2	0.08 (0.006–0.16)	0.036	0.13^∗^ (0.04–0.21)	0.005	0.10 (-0.03–0.22)	0.118	0.04 (-0.08–0.15)	0.548
Multivariable regression analysis model 3	0.09 (0.02–0.16)	0.012	0.13^∗^ (0.05–0.22)	0.003	0.11 (-0.004–0.23)	0.059	0.05 (-0.04–0.15)	0.271

*B = Unstandardized regression coefficient, *CI* = Confidence interval. Model 1: correction for age, sex, educational level, HADS score, and potential vascular risk factors (those that were associated with cognitive decline with *p* < 0.10 in a simple regression model)^∗^. Model 2: correction for age, sex, educational level, HADS score, potential vascular risk factors, and patient group. Model 3: correction for age, sex, educational level, HADS score, potential vascular risk factors, patient group, and baseline cognition. ^∗^Model includes baseline BMI.*

Correlations of determination (*R*^2^’s), indicating the proportions of variance in cognitive decline explained by SVD score or each of the individual MRI markers are shown in **Table 3**. The magnitude of the contributions of the individual markers (as defined in the SVD score) differed across domains, as could be seen from the *R*^2^’s of the individual markers in **Table 3**. The dichotomized WMH explained a smaller proportion of variance in all cognitive domains, except memory, than the SVD score. The proportions of variance in the three cognitive domains explained by the WMH total Fazekas score were comparable to those explained by the SVD score; in overall cognitive decline the WMH total Fazekas score explained a higher proportion of variance than the SVD score.

**Table 3:** *R*^2^ for the association between cognitive decline and small vessel disease (SVD) score versus individual MRI markers.

	Overall cognition; *R*^2^ (*p*)	Executive function; *R*^2^ (*p*)	Information processing speed; *R*^2^ (*p*)	Memory; *R*^2^ (*p*)
SVD score	4.5% (0.017)	13.0% (<0.001)	3.4% (0.037)	0.0% (0.911)
WMH total Fazekas score	7.8% (0.001)	12.9% (<0.001)	4.3% (0.017)	1.0% (0.259)
WMH (dichotomized)^∗^	1.7% (0.139)	6.5% (0.003)	1.6% (0.150)	0.0% (0.938)
Presence of lacunes^∗^	2.1% (0.100)	9.6% (<0.001)	0.0% (0.879)	0.2% (0.590)
Presence of microbleeds^∗^	1.4% (0.180)	1.2% (0.222)	3.9% (0.024)	0.0% (0.816)
Moderate-severe perivascular spaces^∗^	2.5% (0.077)	7.6% (0.002)	3.7% (0.028)	0.4% (0.489)

*R^2^ = Coefficient of determination, SVD = Small vessel disease, WMH = White matter hyperintensities. ^∗^Presence of individual markers were defined as in the SVD score.*

## Discussion

We showed that a total SVD score, that captures total SVD-related brain damage on MRI imaging, was associated with cognitive decline in executive function over 4 years in patients with hypertension.

Previous studies showed associations between cognitive decline and baseline degree of WMH (Prins et al., 2005; Jokinen et al., 2011) or number of lacunes (Prins et al., 2005; Imamine et al., 2011). However, these studies have investigated associations with individual markers of cerebral SVD and no other longitudinal study on cognitive decline considered the total extent of SVD. By capturing all different MRI markers of cerebral SVD in one measure, we think that the SVD score better represents the underlying severity of the disease than just one MRI marker.

Some of the MRI markers of cerebral SVD might be considered more important than others in the association with cognitive function. Our results showed that while the dichotomized WMH explained smaller proportions of variance in cognitive decline compared to the SVD score, the WMH total Fazekas score explained similar or even higher proportions of variance in cognitive decline. This might be explained by a wider range of severity that is captured by the Fazekas scale (score ranging 0–6) compared to the SVD score (ranging 0–4). We believe that the SVD score is an alternative to WMH Fazekas scale and provides a more complete overview of all SVD-related brain damage. We advocate that all of these four markers should be considered together when studying the consequences of SVD-related brain damage.

Besides the four MRI markers of cerebral SVD included in the SVD score, other markers of brain damage might influence cognitive decline, such as cerebral atrophy (Lawrence et al., 2013) or hippocampal volume (O’Brien et al., 2004). Whether cerebral atrophy is part of SVD is still a point of discussion. Cerebral atrophy is not specific for SVD, since it also occurs in normal aging or can be related to focal injury. Atrophy was found to be only marginally associated with SVD score (Staals et al., 2014). However, the unavailability of an atrophy measure in our data should be considered a limitation of our study. In addition, all MRI markers in the SVD score are dichotomized, so degrees of WMH and perivascular spaces or number of lacunes or microbleeds were not accounted for in the score. Future research might test different points in the SVD score for different degrees and thereby may lead to a more optimal SVD score in predicting cognitive decline. Finally, the SVD score includes perivascular spaces in the basal ganglia, but not in the white matter. This can, however, be justified because PVS in the basal ganglia, not those in the white matter, are associated with SVD in terms of lacunar stroke (Doubal et al., 2010; Potter et al., 2015), WMH (Doubal et al., 2010), progression of WMH (Loos et al., 2015), blood pressure (Klarenbeek et al., 2013a), and cognition (Huijts et al., 2014). PVS in the white matter seem to be related to amyloid angiopathy (Charidimou et al., 2015).

The SVD score has been studied in association with cognitive performance before, but these studies were cross-sectional. We published before (Huijts et al., 2013) on a cohort of 189 lacunar stroke and hypertension patients, of which our present study cohort is a subset consisting of those patients who had hypertension and follow-up measurements. In our cross-sectional baseline study, SVD score was related to information processing speed and overall cognition, after correction for age and sex. Another cross-sectional study (Staals et al., 2015) in an age-cohort of 680 older participants showed that the SVD score is associated with general cognitive ability. Additional latent variable modeling analyses was also performed in that study, which showed that the 4 MRI markers are indeed jointly indicative of an underlying overall SVD state.

The finding that only decline in executive function is associated with the SVD score might indicate an expression of subcortical damage caused by SVD. The result is in line with a recent study (Lawrence et al., 2015) in patients with lacunar stroke and leukoaraiosis, in which patients showed a significant decline in executive function, but not in other domains (processing speed, working memory, and global cognition).

A major strength is the longitudinal design of our study with a 4-year follow-up period. In addition, all patients received a comprehensive neuropsychological assessment, which included multiple tests in three cognitive domains. A limitation of the study is the use of variable MRI field strength. Therefore, we performed sub-analyses including only the patients who were scanned at 1.5T (*n* = 109). This showed not only significant associations between the SVD score and cognitive decline in overall cognition and executive functioning, but also with information processing speed, when we used the same variables as in multivariable regression model 3 (results not shown). The second limitation is the mixed hypertensive population. To reach a relatively large sample with a broad spectrum of severity of cerebral SVD, we included both hypertensive patients with and without a small vessel stroke. Stroke itself might have influenced cognition. However, the inclusion of patient group as a confounder in the model did not change the results. In addition, three lacunar stroke patients had a recurrent stroke during follow-up, which might have influenced cognitive decline. However, results did not change when we excluded these three patients from analyses (results not shown). Still, we studied a relatively healthy population; a large part of the patient group had a SVD score of 0. This might reduce the generalizability of the results to more affected patient populations. In addition, results may differ in other SVD populations, such as in SVD patients with intracerebral hemorrhage. Lastly, due to selection bias cognitively healthier people might be included in the study at baseline, which might have led to an underestimation of the reported associations. Future studies are needed to investigate the association between the SVD score and cognitive decline in a more affected and more homogeneous population.

This longitudinal study investigated a combined score of SVD MRI markers in relation to cognitive decline and shows that this score predicts future cognitive decline in executive functioning. The SVD score might be a good alternative to the Fazekas WMH score in predicting cognitive decline, since the SVD score might provide a more complete overview of total SVD-related brain damage.

## Author Contributions

Conception and design of the research: RU, RO, MH, PL, AK, and JS. Acquisition of the data: RU and MH. Analysis and interpretation of the data: RU, RO, and JS. Drafting the manuscript: RU, RO, and JS. Critical revision of the manuscript: RU, RO, MH, PL, AK, and JS. Final approval of the version to be published: RU, RO, MH, PL, AK, and JS.

## Conflict of Interest Statement

The authors declare that the research was conducted in the absence of any commercial or financial relationships that could be construed as a potential conflict of interest.
