# Supplementary material for: Total Cerebral Small Vessel Disease MRI Score Is Associated with Cognitive Decline in Executive Function in Patients with Hypertension
Source: Front Aging Neurosci. 2016 Dec 12;8:301. doi: 10.3389/fnagi.2016.00301 (PMC5149514; doi:10.3389/fnagi.2016.00301)
Supplement: Supplementary file 1 [file Data_Sheet_1.DOCX]

Supplementary Material

**Total Cerebral Small Vessel Disease MRI Score Is Associated With Cognitive Decline In Executive Function In Patients With Hypertension**

**Renske Uiterwijk^1, 4^*, Robert J. van Oostenbrugge^1, 4, 5^, Marjolein Huijts^2^, Peter W. De Leeuw^3, 5^, Abraham A. Kroon^3, 5^, Julie Staals^1, 5^**

*** Correspondence:** Renske Uiterwijk: renske.uiterwijk@mumc.nl

**Supplementary MRI data**

MRI sequence parameters for the uncomplicated hypertensive patients (all patients were scanned on a 1.5T MRI scanner, Philips Medical Systems, Best, The Netherlands):

T2-weighted turbo spin echo sequence (TR/TE = 4820/100 ms), T2*-weighted gradient echo sequence (TR/TE=736/23 ms), and FLAIR sequence (TR/TI/TE=8000/2000/120), all with thickness of 5 mm and 0.5mm interslice gap.

MRI sequence parameters for the hypertensive lacunar stroke patients: (18 patients were scanned on a 1.5T and 21 patients on a 3T MRI scanner, both scanners: Philips Medical Systems, Best, The Netherlands).

1.5T: T2-weighted turbo spin echo sequence (TR/TE = shortest(5515)/100 ms), T2*-weighted gradient echo sequence (TR/TE = shortest(851)/23 ms) and FLAIR sequence (TR/TI/TE = 8000/2000/120 ms), all with slice thickness 5mm and 0.5mm interslice gap.

3.0T: T2-weighted turbo spin echo sequence (TR/TE = 3000/80 ms), T2*-weighted gradient echo sequence (TR/TE = shortest(794)/16 ms) and FLAIR sequence (TR/TI/TE =11000/2800/125 ms), all with slice thickness 5mm and 0.5mm interslice gap.
